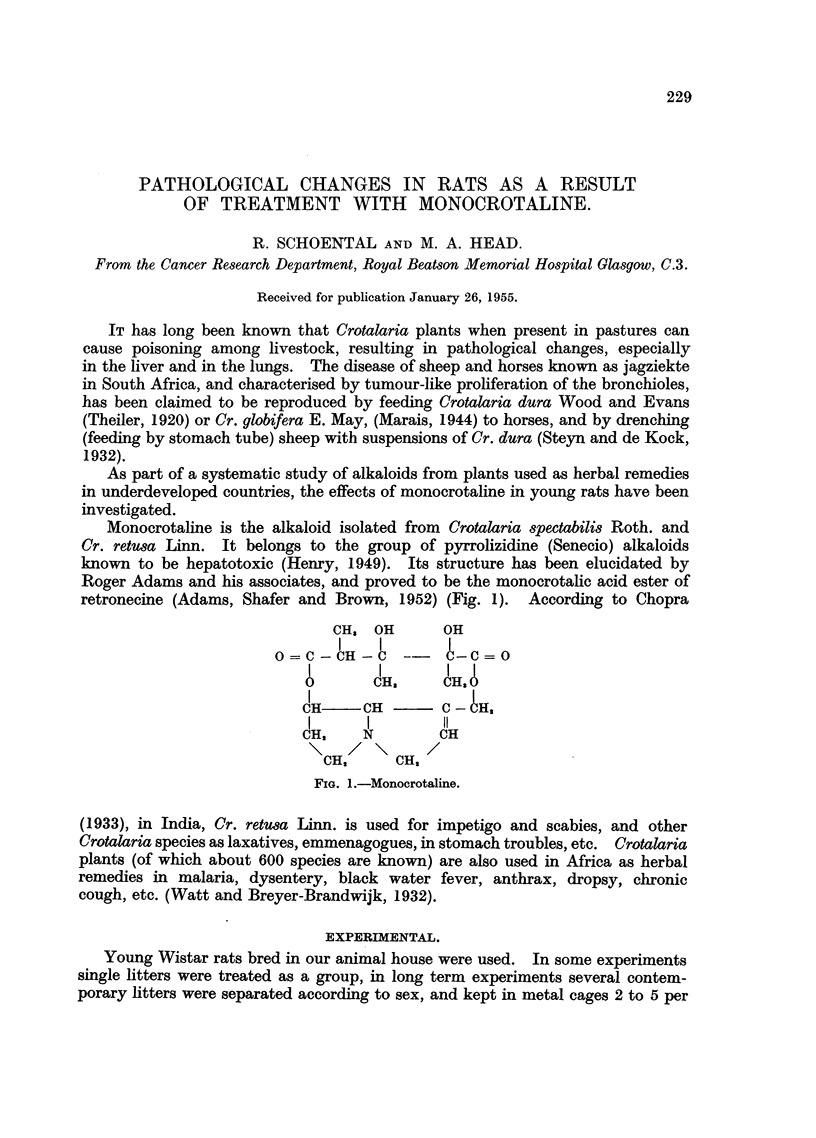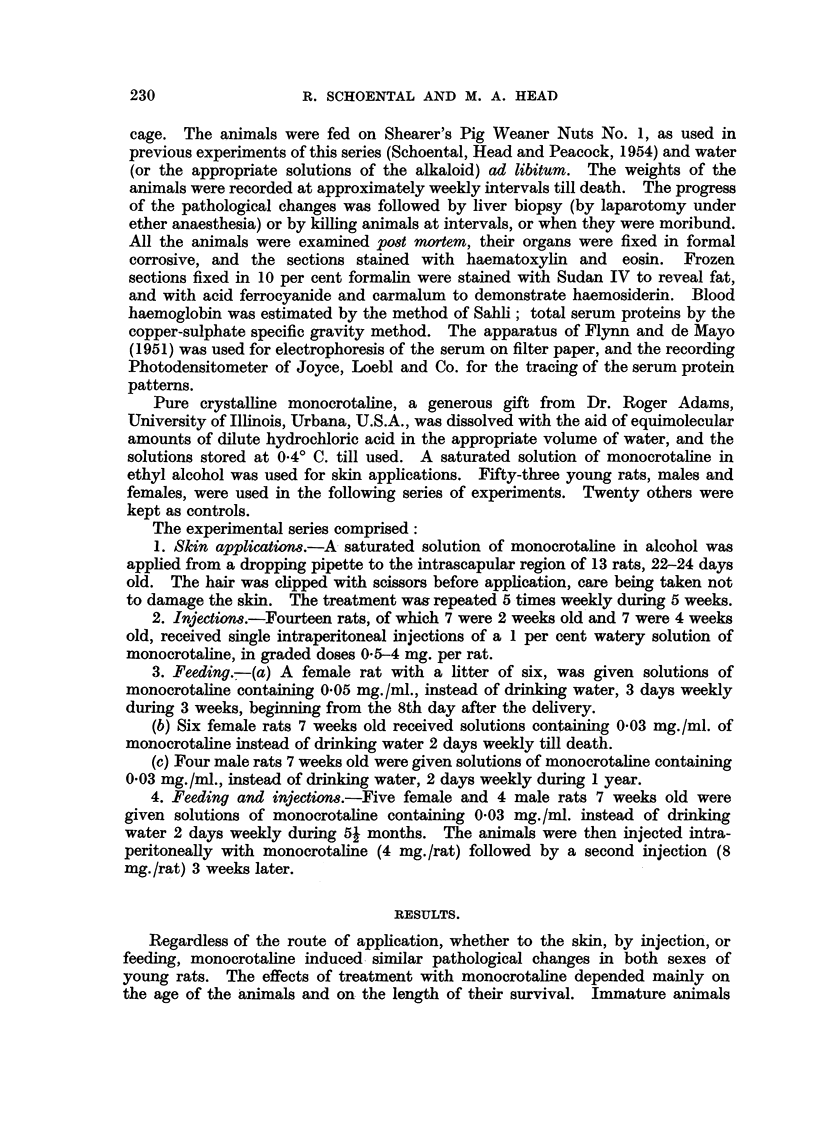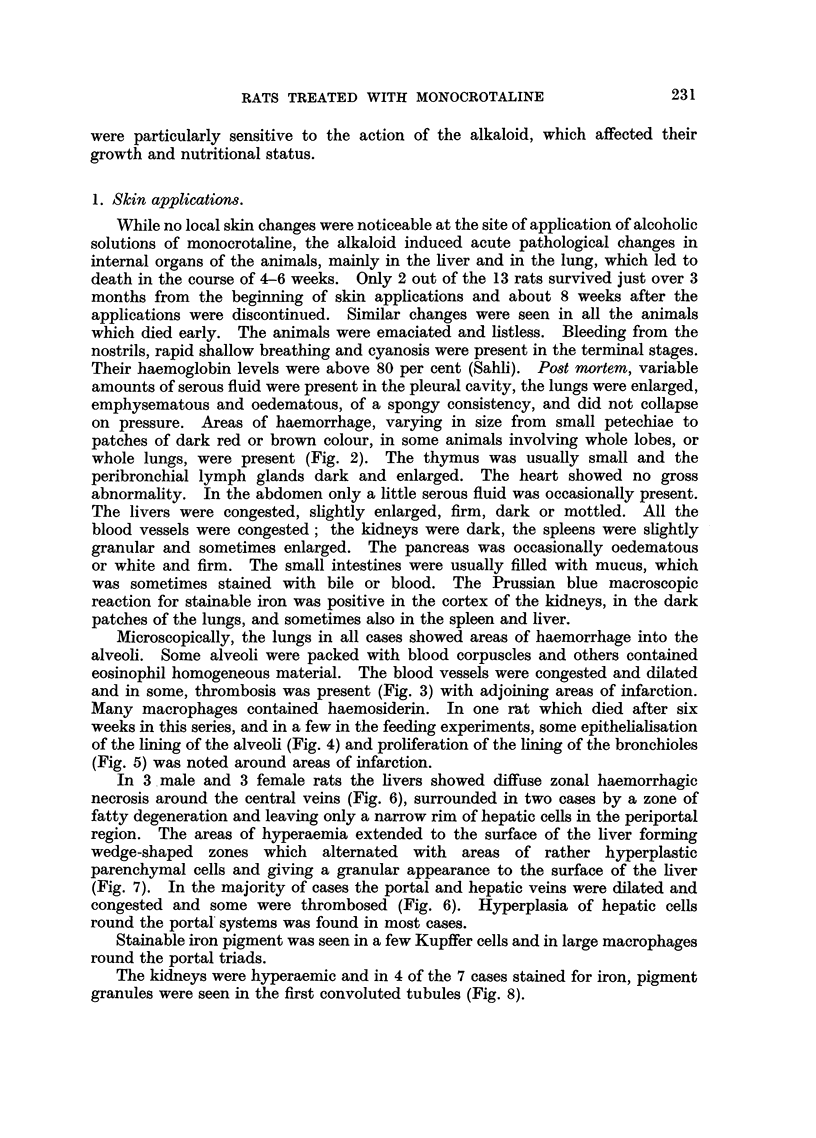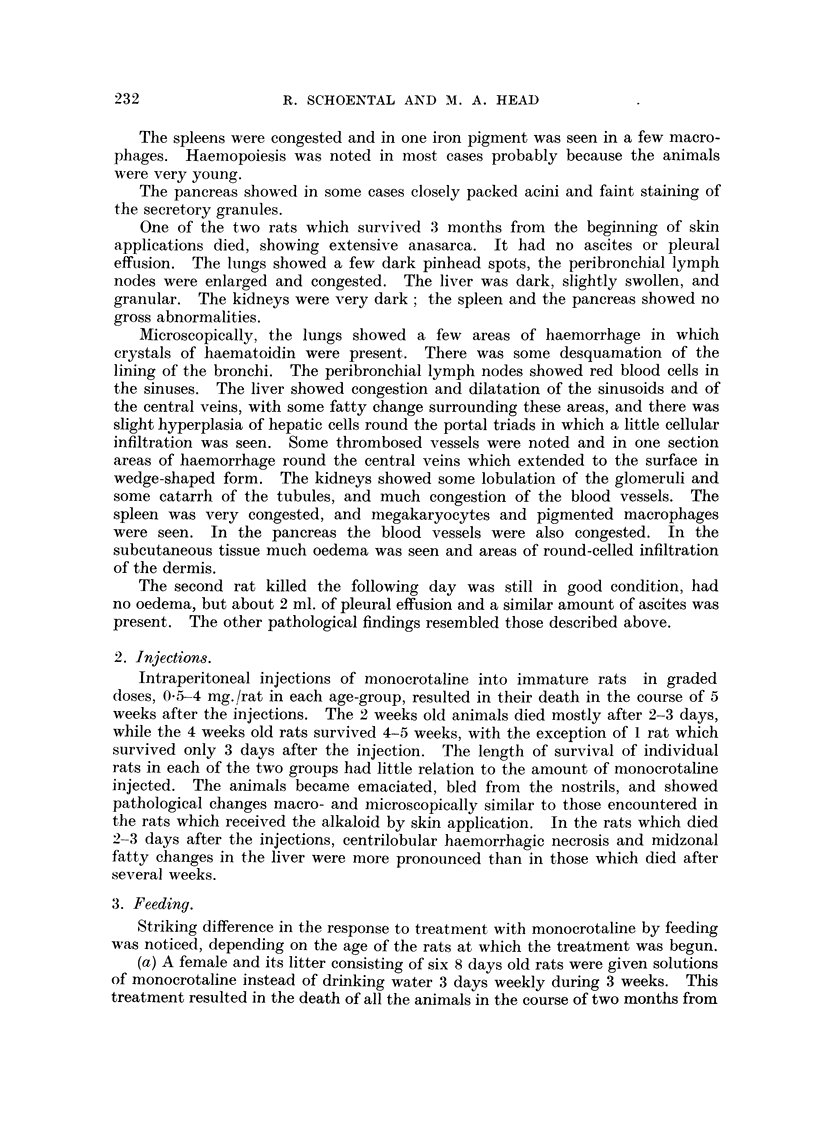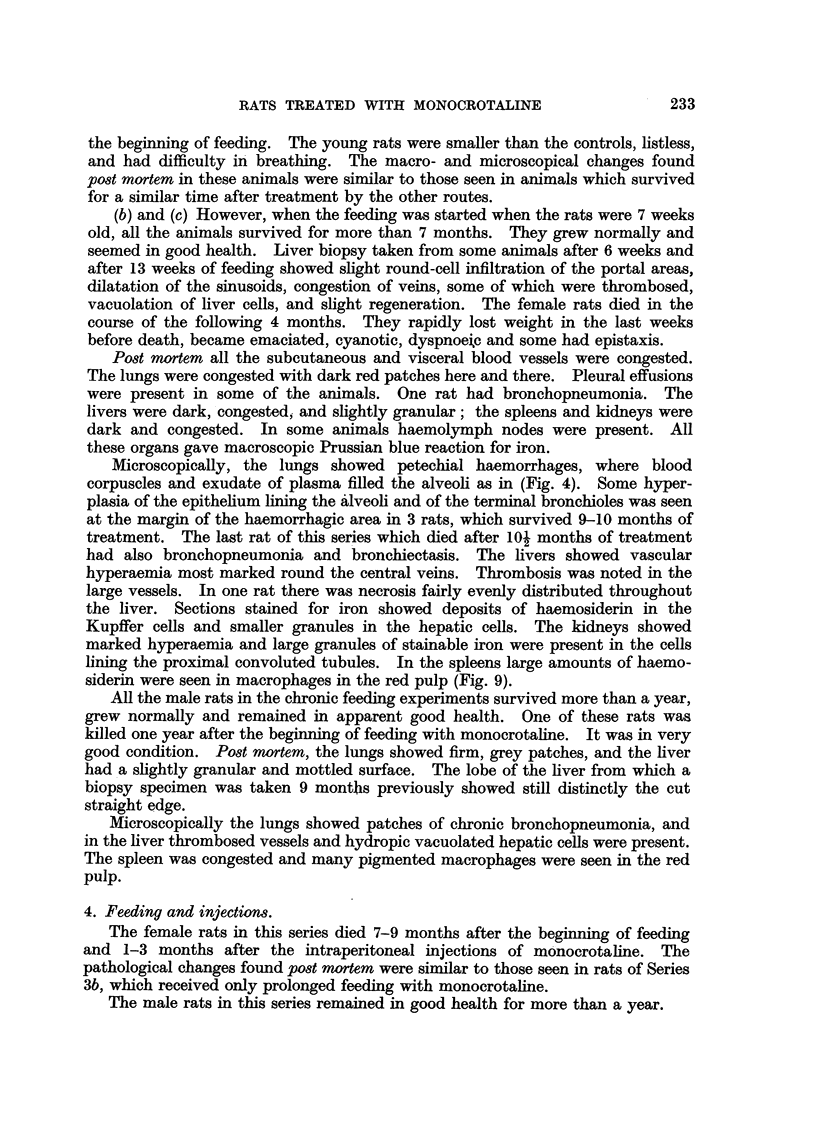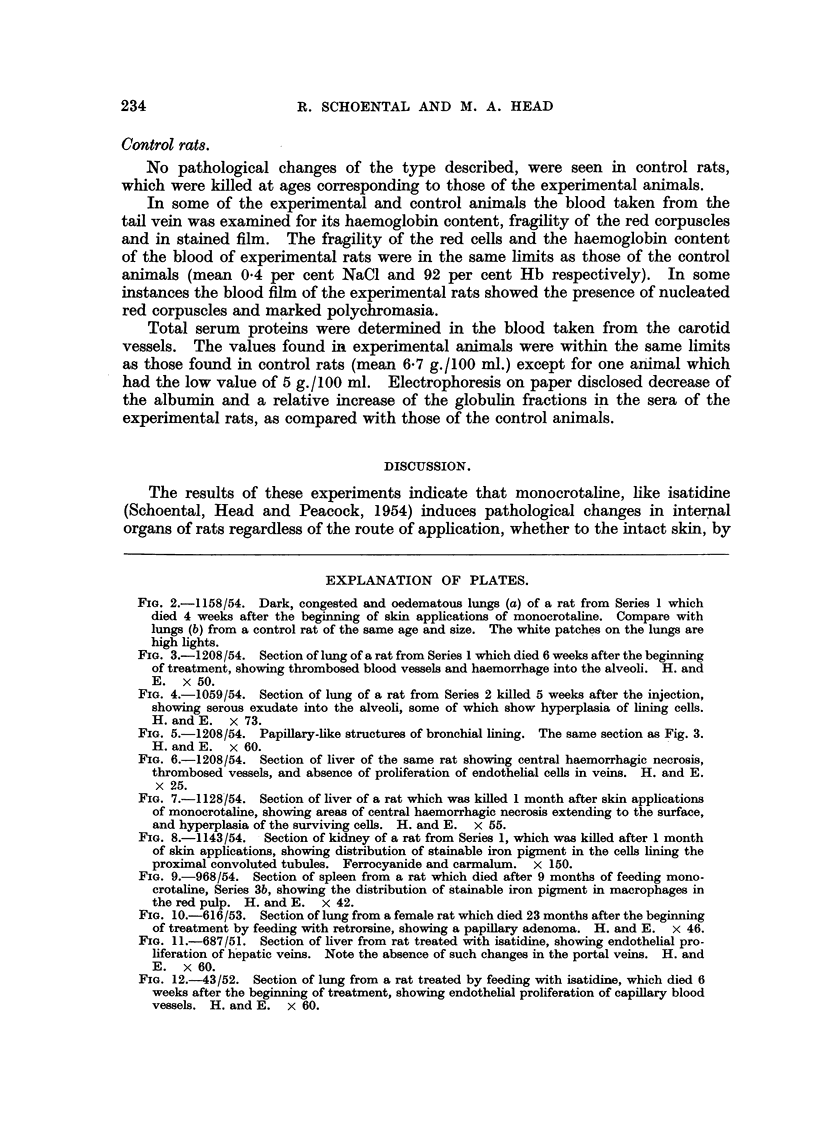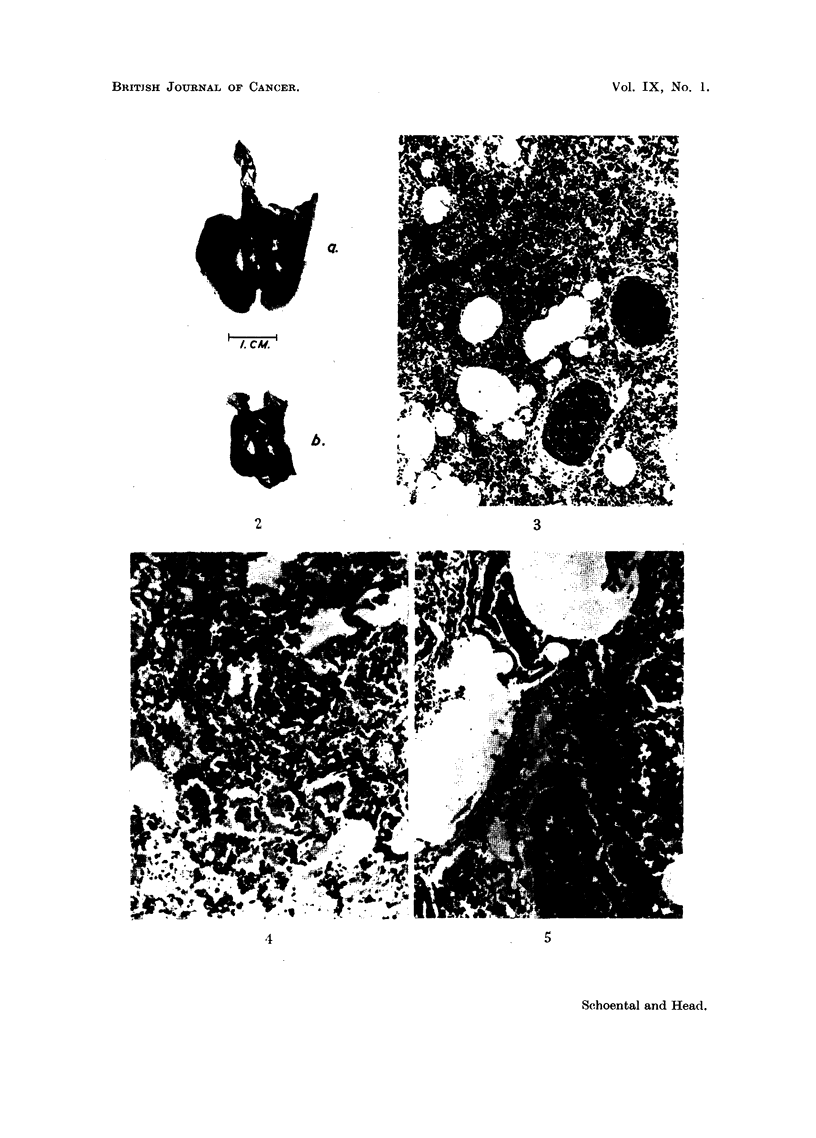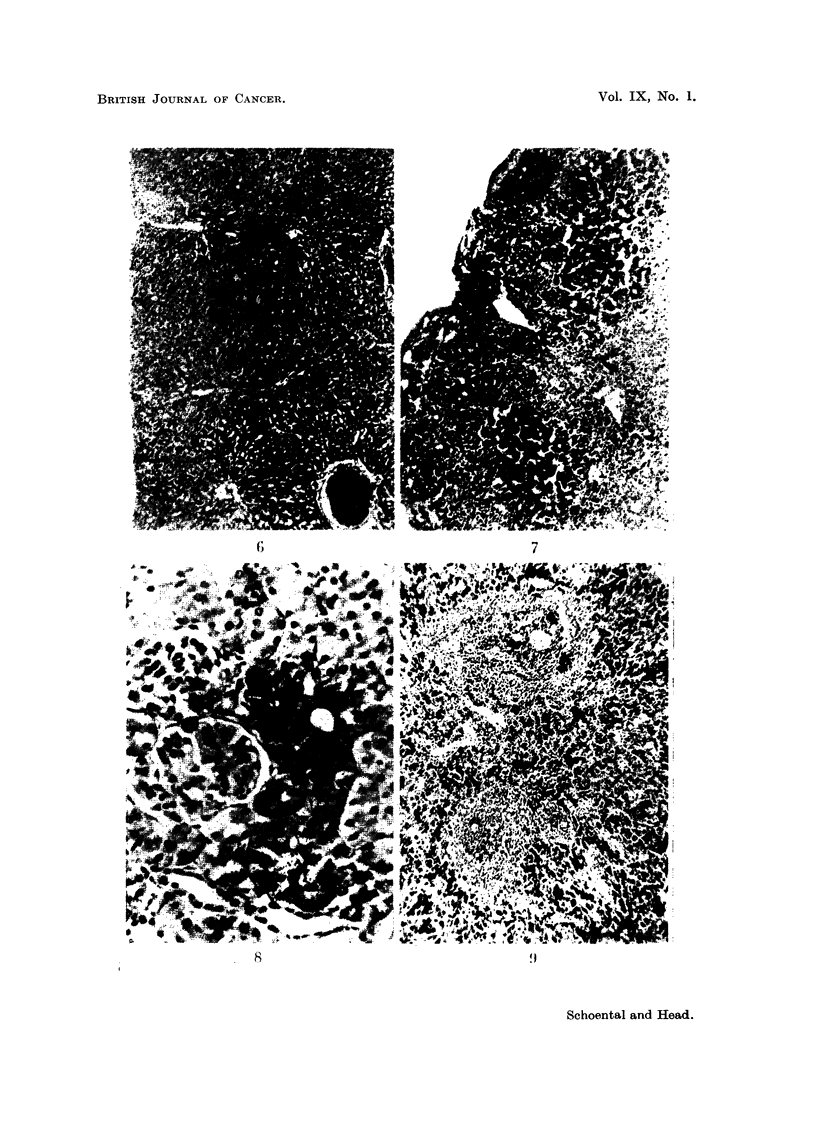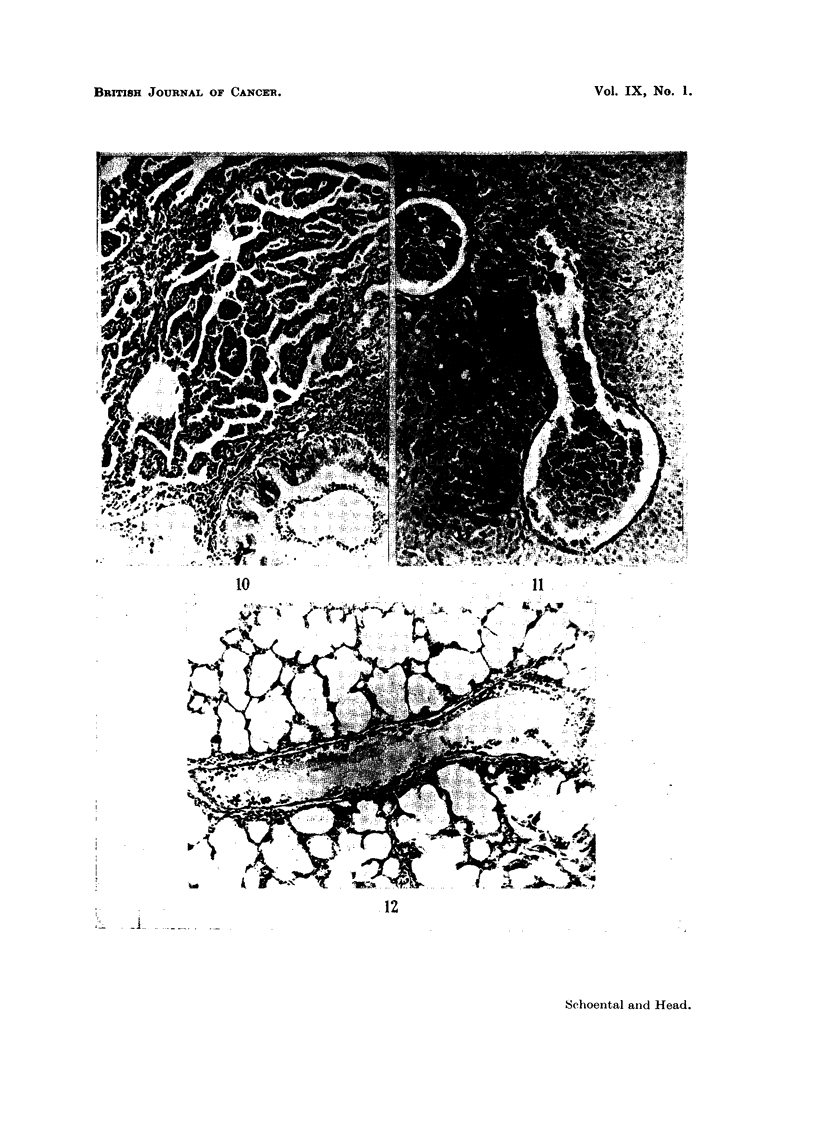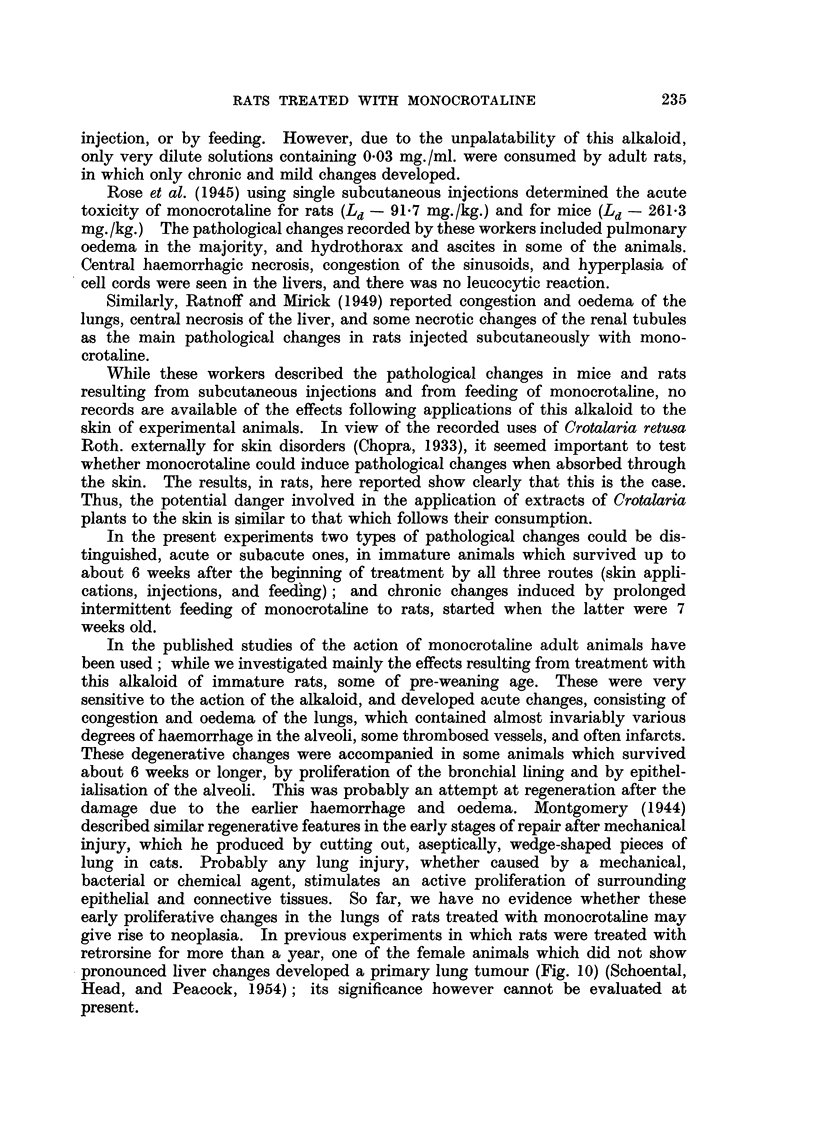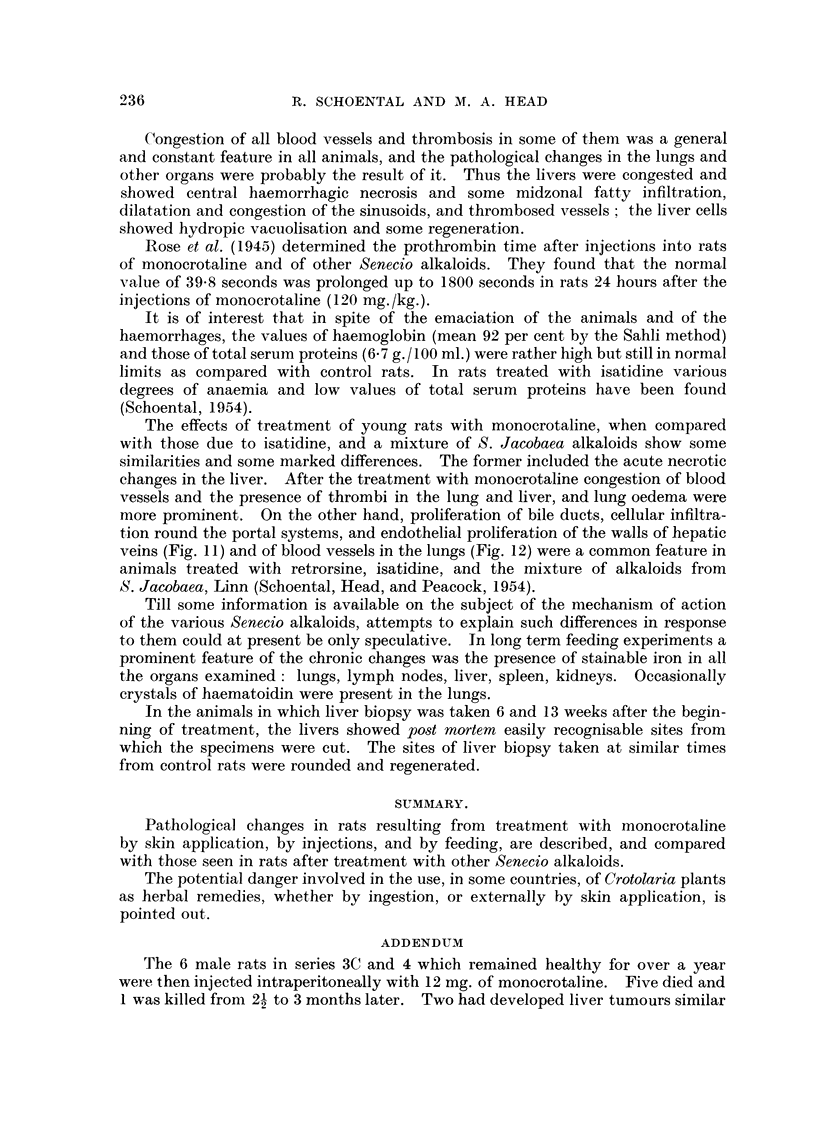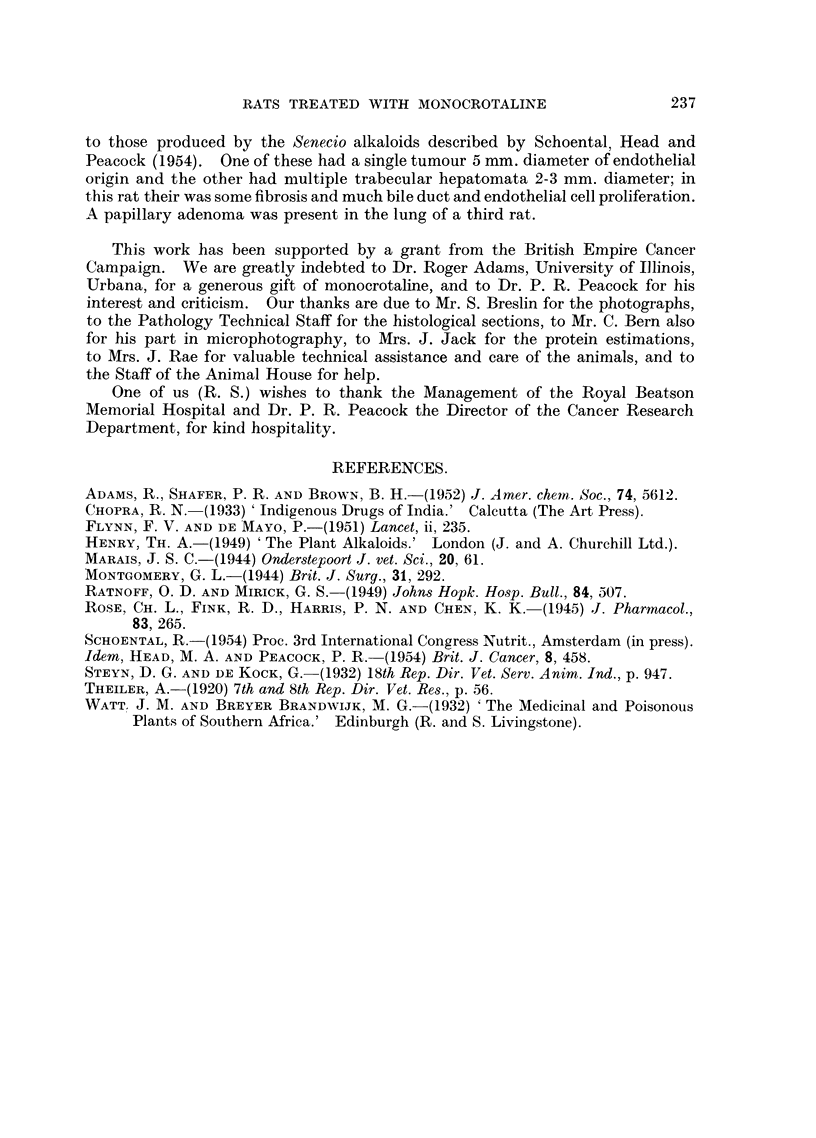# Pathological Changes in Rats as a Result of Treatment with Monocrotaline

**DOI:** 10.1038/bjc.1955.19

**Published:** 1955-03

**Authors:** R. Schoental, M. A. Head

## Abstract

**Images:**


### PATHOLOGICAL CHANGES IN RATS AS A RESULT OF TREATMENT WITH MONOCROTALINE.

R. SCHOENTAL AND M. A. HEAD.

*From the Cancer Research Department, Royal Beatson Memorial Hospital Glasgow, C.3.*

Received for publication January 26, 1955.

IT has long been known that *Crotalaria* plants when present in pastures can cause poisoning among livestock, resulting in pathological changes, especially in the liver and in the lungs. The disease of sheep and horses known as jagziekte in South Africa, and characterised by tumour-like proliferation of the bronchioles, has been claimed to be reproduced by feeding *Crotalaria dura* Wood and Evans (Theiler, 1920) or *Cr. globifera* E. May, (Marais, 1944) to horses, and by drenching (feeding by stomach tube) sheep with suspensions of *Cr. dura* (Steyn and de Kock, 1932).

As part of a systematic study of alkaloids from plants used as herbal remedies in underdeveloped countries, the effects of monocrotaline in young rats have been investigated.

Monocrotaline is the alkaloid isolated from *Crotalaria spectabilis* Roth. and *Cr. retusa* Linn. It belongs to the group of pyrrolizidine (Senecio) alkaloids known to be hepatotoxic (Henry, 1949). Its structure has been elucidated by Roger Adams and his associates, and proved to be the monocrotalic acid ester of retronecine (Adams, Shafer and Brown, 1952) (Fig. 1). According to Chopra

FIG. 1.—Monocrotaline.

(1933), in India, *Cr. retusa* Linn. is used for impetigo and scabies, and other *Crotalaria* species as laxatives, emmenagogues, in stomach troubles, etc. *Crotalaria* plants (of which about 600 species are known) are also used in Africa as herbal remedies in malaria, dysentery, black water fever, anthrax, dropsy, chronic cough, etc. (Watt and Breyer-Brandwijk, 1932).

### EXPERIMENTAL.

Young Wistar rats bred in our animal house were used. In some experiments single litters were treated as a group, in long term experiments several contemporary litters were separated according to sex, and kept in metal cages 2 to 5 per cage. The animals were fed on Shearer's Pig Weaner Nuts No. 1, as used in previous experiments of this series (Schoental, Head and Peacock, 1954) and water (or the appropriate solutions of the alkaloid) *ad libitum.* The weights of the animals were recorded at approximately weekly intervals till death. The progress of the pathological changes was followed by liver biopsy (by laparotomy under ether anaesthesia) or by killing animals at intervals, or when they were moribund. All the animals were examined *post mortem*, their organs were fixed in formal corrosive, and the sections stained with haematoxylin and eosin. Frozen sections fixed in 10 per cent formalin were stained with Sudan IV to reveal fat, and with acid ferrocyanide and carmalum to demonstrate haemosiderin. Blood haemoglobin was estimated by the method of Sahli ; total serum proteins by the copper-sulphate specific gravity method. The apparatus of Flynn and de Mayo (1951) was used for electrophoresis of the serum on filter paper, and the recording Photodensitometer of Joyce, Loebl and Co. for the tracing of the serum protein patterns.

Pure crystalline monocrotaline, a generous gift from Dr. Roger Adams, University of Illinois, Urbana, U.S.A., was dissolved with the aid of equimolecular amounts of dilute hydrochloric acid in the appropriate volume of water, and the solutions stored at 0·4° C. till used. A saturated solution of monocrotaline in ethyl alcohol was used for skin applications. Fifty-three young rats, males and females, were used in the following series of experiments. Twenty others were kept as controls.

The experimental series comprised :

1. *Skin applications.*—A saturated solution of monocrotaline in alcohol was applied from a dropping pipette to the intrascapular region of 13 rats, 22–24 days old. The hair was clipped with scissors before application, care being taken not to damage the skin. The treatment was repeated 5 times weekly during 5 weeks.

2. *Injections.*—Fourteen rats, of which 7 were 2 weeks old and 7 were 4 weeks old, received single intraperitoneal injections of a 1 per cent watery solution of monocrotaline, in graded doses 0·5–4 mg. per rat.

3. *Feeding.*—(*a*) A female rat with a litter of six, was given solutions of monocrotaline containing 0·05 mg./ml., instead of drinking water, 3 days weekly during 3 weeks, beginning from the 8th day after the delivery.

(*b*) Six female rats 7 weeks old received solutions containing 0·03 mg./ml. of monocrotaline instead of drinking water 2 days weekly till death.

(*c*) Four male rats 7 weeks old were given solutions of monocrotaline containing 0·03 mg./ml., instead of drinking water, 2 days weekly during 1 year.

4. *Feeding and injections.*—Five female and 4 male rats 7 weeks old were given solutions of monocrotaline containing 0·03 mg./ml. instead of drinking water 2 days weekly during 5½ months. The animals were then injected intraperitoneally with monocrotaline (4 mg./rat) followed by a second injection (8 mg./rat) 3 weeks later.

### RESULTS.

Regardless of the route of application, whether to the skin, by injection, or feeding, monocrotaline induced similar pathological changes in both sexes of young rats. The effects of treatment with monocrotaline depended mainly on the age of the animals and on the length of their survival. Immature animals were particularly sensitive to the action of the alkaloid, which affected their growth and nutritional status.

1. *Skin applications.*

While no local skin changes were noticeable at the site of application of alcoholic solutions of monocrotaline, the alkaloid induced acute pathological changes in internal organs of the animals, mainly in the liver and in the lung, which led to death in the course of 4–6 weeks. Only 2 out of the 13 rats survived just over 3 months from the beginning of skin applications and about 8 weeks after the applications were discontinued. Similar changes were seen in all the animals which died early. The animals were emaciated and listless. Bleeding from the nostrils, rapid shallow breathing and cyanosis were present in the terminal stages. Their haemoglobin levels were above 80 per cent (Sahli). *Post mortem*, variable amounts of serous fluid were present in the pleural cavity, the lungs were enlarged, emphysematous and oedematous, of a spongy consistency, and did not collapse on pressure. Areas of haemorrhage, varying in size from small petechiae to patches of dark red or brown colour, in some animals involving whole lobes, or whole lungs, were present (Fig. 2). The thymus was usually small and the peribronchial lymph glands dark and enlarged. The heart showed no gross abnormality. In the abdomen only a little serous fluid was occasionally present. The livers were congested, slightly enlarged, firm, dark or mottled. All the blood vessels were congested ; the kidneys were dark, the spleens were slightly granular and sometimes enlarged. The pancreas was occasionally oedematous or white and firm. The small intestines were usually filled with mucus, which was sometimes stained with bile or blood. The Prussian blue macroscopic reaction for stainable iron was positive in the cortex of the kidneys, in the dark patches of the lungs, and sometimes also in the spleen and liver.

Microscopically, the lungs in all cases showed areas of haemorrhage into the alveoli. Some alveoli were packed with blood corpuscles and others contained eosinophil homogeneous material. The blood vessels were congested and dilated and in some, thrombosis was present (Fig. 3) with adjoining areas of infarction. Many macrophages contained haemosiderin. In one rat which died after six weeks in this series, and in a few in the feeding experiments, some epithelialisation of the lining of the alveoli (Fig. 4) and proliferation of the lining of the bronchioles (Fig. 5) was noted around areas of infarction.

In 3 male and 3 female rats the livers showed diffuse zonal haemorrhagic necrosis around the central veins (Fig. 6), surrounded in two cases by a zone of fatty degeneration and leaving only a narrow rim of hepatic cells in the periportal region. The areas of hyperaemia extended to the surface of the liver forming wedge-shaped zones which alternated with areas of rather hyperplastic parenchymal cells and giving a granular appearance to the surface of the liver (Fig. 7). In the majority of cases the portal and hepatic veins were dilated and congested and some were thrombosed (Fig. 6). Hyperplasia of hepatic cells round the portal systems was found in most cases.

Stainable iron pigment was seen in a few Kupffer cells and in large macrophages round the portal triads.

The kidneys were hyperaemic and in 4 of the 7 cases stained for iron, pigment granules were seen in the first convoluted tubules (Fig. 8).

The spleens were congested and in one iron pigment was seen in a few macrophages. Haemopoiesis was noted in most cases probably because the animals were very young.

The pancreas showed in some cases closely packed acini and faint staining of the secretory granules.

One of the two rats which survived 3 months from the beginning of skin applications died, showing extensive anasarca. It had no ascites or pleural effusion. The lungs showed a few dark pinhead spots, the peribronchial lymph nodes were enlarged and congested. The liver was dark, slightly swollen, and granular. The kidneys were very dark ; the spleen and the pancreas showed no gross abnormalities.

Microscopically, the lungs showed a few areas of haemorrhage in which crystals of haematoidin were present. There was some desquamation of the lining of the bronchi. The peribronchial lymph nodes showed red blood cells in the sinuses. The liver showed congestion and dilatation of the sinusoids and of the central veins, with some fatty change surrounding these areas, and there was slight hyperplasia of hepatic cells round the portal triads in which a little cellular infiltration was seen. Some thrombosed vessels were noted and in one section areas of haemorrhage round the central veins which extended to the surface in wedge-shaped form. The kidneys showed some lobulation of the glomeruli and some catarrh of the tubules, and much congestion of the blood vessels. The spleen was very congested, and megakaryocytes and pigmented macrophages were seen. In the pancreas the blood vessels were also congested. In the subcutaneous tissue much oedema was seen and areas of round-celled infiltration of the dermis.

The second rat killed the following day was still in good condition, had no oedema, but about 2 ml. of pleural effusion and a similar amount of ascites was present. The other pathological findings resembled those described above.

2. *Injections.*

Intraperitoneal injections of monocrotaline into immature rats in graded doses, 0·5–4 mg./rat in each age-group, resulted in their death in the course of 5 weeks after the injections. The 2 weeks old animals died mostly after 2–3 days, while the 4 weeks old rats survived 4–5 weeks, with the exception of 1 rat which survived only 3 days after the injection. The length of survival of individual rats in each of the two groups had little relation to the amount of monocrotaline injected. The animals became emaciated, bled from the nostrils, and showed pathological changes macro- and microscopically similar to those encountered in the rats which received the alkaloid by skin application. In the rats which died 2–3 days after the injections, centrilobular haemorrhagic necrosis and midzonal fatty changes in the liver were more pronounced than in those which died after several weeks.

3. *Feeding.*

Striking difference in the response to treatment with monocrotaline by feeding was noticed, depending on the age of the rats at which the treatment was begun.

(*a*) A female and its litter consisting of six 8 days old rats were given solutions of monocrotaline instead of drinking water 3 days weekly during 3 weeks. This treatment resulted in the death of all the animals in the course of two months from the beginning of feeding. The young rats were smaller than the controls, listless, and had difficulty in breathing. The macro- and microscopical changes found *post mortem* in these animals were similar to those seen in animals which survived for a similar time after treatment by the other routes.

(*b*) and (*c*) However, when the feeding was started when the rats were 7 weeks old, all the animals survived for more than 7 months. They grew normally and seemed in good health. Liver biopsy taken from some animals after 6 weeks and after 13 weeks of feeding showed slight round-cell infiltration of the portal areas, dilatation of the sinusoids, congestion of veins, some of which were thrombosed, vacuolation of liver cells, and slight regeneration. The female rats died in the course of the following 4 months. They rapidly lost weight in the last weeks before death, became emaciated, cyanotic, dyspnoeic and some had epistaxis.

*Post mortem* all the subcutaneous and visceral blood vessels were congested. The lungs were congested with dark red patches here and there. Pleural effusions were present in some of the animals. One rat had bronchopneumonia. The livers were dark, congested, and slightly granular; the spleens and kidneys were dark and congested. In some animals haemolymph nodes were present. All these organs gave macroscopic Prussian blue reaction for iron.

Microscopically, the lungs showed petechial haemorrhages, where blood corpuscles and exudate of plasma filled the alveoli as in (Fig. 4). Some hyperplasia of the epithelium lining the alveoli and of the terminal bronchioles was seen at the margin of the haemorrhagic area in 3 rats, which survived 9–10 months of treatment. The last rat of this series which died after 10½ months of treatment had also bronchopneumonia and bronchiectasis. The livers showed vascular hyperaemia most marked round the central veins. Thrombosis was noted in the large vessels. In one rat there was necrosis fairly evenly distributed throughout the liver. Sections stained for iron showed deposits of haemosiderin in the Kupffer cells and smaller granules in the hepatic cells. The kidneys showed marked hyperaemia and large granules of stainable iron were present in the cells lining the proximal convoluted tubules. In the spleens large amounts of haemosiderin were seen in macrophages in the red pulp (Fig. 9).

All the male rats in the chronic feeding experiments survived more than a year, grew normally and remained in apparent good health. One of these rats was killed one year after the beginning of feeding with monocrotaline. It was in very good condition. *Post mortem*, the lungs showed firm, grey patches, and the liver had a slightly granular and mottled surface. The lobe of the liver from which a biopsy specimen was taken 9 months previously showed still distinctly the cut straight edge.

Microscopically the lungs showed patches of chronic bronchopneumonia, and in the liver thrombosed vessels and hydropic vacuolated hepatic cells were present. The spleen was congested and many pigmented macrophages were seen in the red pulp.

4. *Feeding and injections.*

The female rats in this series died 7–9 months after the beginning of feeding and 1–3 months after the intraperitoneal injections of monocrotaline. The pathological changes found *post mortem* were similar to those seen in rats of Series 3*b*, which received only prolonged feeding with monocrotaline.

The male rats in this series remained in good health for more than a year.

*Control rats.*

No pathological changes of the type described, were seen in control rats, which were killed at ages corresponding to those of the experimental animals.

In some of the experimental and control animals the blood taken from the tail vein was examined for its haemoglobin content, fragility of the red corpuscles and in stained film. The fragility of the red cells and the haemoglobin content of the blood of experimental rats were in the same limits as those of the control animals (mean 0·4 per cent NaCl and 92 per cent Hb respectively). In some instances the blood film of the experimental rats showed the presence of nucleated red corpuscles and marked polychromasia.

Total serum proteins were determined in the blood taken from the carotid vessels. The values found in experimental animals were within the same limits as those found in control rats (mean 6·7 g./100 ml.) except for one animal which had the low value of 5 g./100 ml. Electrophoresis on paper disclosed decrease of the albumin and a relative increase of the globulin fractions in the sera of the experimental rats, as compared with those of the control animals.

### DISCUSSION.

The results of these experiments indicate that monocrotaline, like isatidine (Schoental, Head and Peacock, 1954) induces pathological changes in internal organs of rats regardless of the route of application, whether to the intact skin, by

---

### EXPLANATION OF PLATES.

FIG. 2.—1158/54. Dark, congested and oedematous lungs (*a*) of a rat from Series 1 which died 4 weeks after the beginning of skin applications of monocrotaline. Compare with lungs (*b*) from a control rat of the same age and size. The white patches on the lungs are high lights.

FIG. 3.—1208/54. Section of lung of a rat from Series 1 which died 6 weeks after the beginning of treatment, showing thrombosed blood vessels and haemorrhage into the alveoli. H. and E. × 50.

FIG. 4.—1059/54. Section of lung of a rat from Series 2 killed 5 weeks after the injection, showing serous exudate into the alveoli, some of which show hyperplasia of lining cells. H. and E. × 73.

FIG. 5.—1208/54. Papillary-like structures of bronchial lining. The same section as Fig. 3. H. and E. × 60.

FIG. 6.—1208/54. Section of liver of the same rat showing central haemorrhagic necrosis, thrombosed vessels, and absence of proliferation of endothelial cells in veins. H. and E. × 25.

FIG. 7.—1128/54. Section of liver of a rat which was killed 1 month after skin applications of monocrotaline, showing areas of central haemorrhagic necrosis extending to the surface, and hyperplasia of the surviving cells. H. and E. × 55.

FIG. 8.—1143/54. Section of kidney of a rat from Series 1, which was killed after 1 month of skin applications, showing distribution of stainable iron pigment in the cells lining the proximal convoluted tubules. Ferrocyanide and carmalum. × 150.

FIG. 9.—968/54. Section of spleen from a rat which died after 9 months of feeding monocrotaline, Series 3*b*, showing the distribution of stainable iron pigment in macrophages in the red pulp. H. and E. × 42.

FIG. 10.—616/53. Section of lung from a female rat which died 23 months after the beginning of treatment by feeding with retrorsine, showing a papillary adenoma. H. and E. × 46.

FIG. 11.—687/51. Section of liver from rat treated with isatidine, showing endothelial proliferation of hepatic veins. Note the absence of such changes in the portal veins. H. and E. × 60.

FIG. 12.—43/52. Section of lung from a rat treated by feeding with isatidine, which died 6 weeks after the beginning of treatment, showing endothelial proliferation of capillary blood vessels. H. and E. × 60.

2

3

4

5

Schoental and Head.

6

7

8

9

Schoental and Head.

10

11

12

injection, or by feeding. However, due to the unpalatability of this alkaloid, only very dilute solutions containing 0·03 mg./ml. were consumed by adult rats, in which only chronic and mild changes developed.

Rose *et al.* (1945) using single subcutaneous injections determined the acute toxicity of monocrotaline for rats ($L_d$ — 91·7 mg./kg.) and for mice ($L_d$ — 261·3 mg./kg.) The pathological changes recorded by these workers included pulmonary oedema in the majority, and hydrothorax and ascites in some of the animals. Central haemorrhagic necrosis, congestion of the sinusoids, and hyperplasia of cell cords were seen in the livers, and there was no leucocytic reaction.

Similarly, Ratnoff and Mirick (1949) reported congestion and oedema of the lungs, central necrosis of the liver, and some necrotic changes of the renal tubules as the main pathological changes in rats injected subcutaneously with monocrotaline.

While these workers described the pathological changes in mice and rats resulting from subcutaneous injections and from feeding of monocrotaline, no records are available of the effects following applications of this alkaloid to the skin of experimental animals. In view of the recorded uses of *Crotalaria retusa* Roth. externally for skin disorders (Chopra, 1933), it seemed important to test whether monocrotaline could induce pathological changes when absorbed through the skin. The results, in rats, here reported show clearly that this is the case. Thus, the potential danger involved in the application of extracts of *Crotalaria* plants to the skin is similar to that which follows their consumption.

In the present experiments two types of pathological changes could be distinguished, acute or subacute ones, in immature animals which survived up to about 6 weeks after the beginning of treatment by all three routes (skin applications, injections, and feeding); and chronic changes induced by prolonged intermittent feeding of monocrotaline to rats, started when the latter were 7 weeks old.

In the published studies of the action of monocrotaline adult animals have been used; while we investigated mainly the effects resulting from treatment with this alkaloid of immature rats, some of pre-weaning age. These were very sensitive to the action of the alkaloid, and developed acute changes, consisting of congestion and oedema of the lungs, which contained almost invariably various degrees of haemorrhage in the alveoli, some thrombosed vessels, and often infarcts. These degenerative changes were accompanied in some animals which survived about 6 weeks or longer, by proliferation of the bronchial lining and by epithelialisation of the alveoli. This was probably an attempt at regeneration after the damage due to the earlier haemorrhage and oedema. Montgomery (1944) described similar regenerative features in the early stages of repair after mechanical injury, which he produced by cutting out, aseptically, wedge-shaped pieces of lung in cats. Probably any lung injury, whether caused by a mechanical, bacterial or chemical agent, stimulates an active proliferation of surrounding epithelial and connective tissues. So far, we have no evidence whether these early proliferative changes in the lungs of rats treated with monocrotaline may give rise to neoplasia. In previous experiments in which rats were treated with retrorsine for more than a year, one of the female animals which did not show pronounced liver changes developed a primary lung tumour (Fig. 10) (Schoental, Head, and Peacock, 1954); its significance however cannot be evaluated at present.

Congestion of all blood vessels and thrombosis in some of them was a general and constant feature in all animals, and the pathological changes in the lungs and other organs were probably the result of it. Thus the livers were congested and showed central haemorrhagic necrosis and some midzonal fatty infiltration, dilatation and congestion of the sinusoids, and thrombosed vessels ; the liver cells showed hydropic vacuolisation and some regeneration.

Rose *et al.* (1945) determined the prothrombin time after injections into rats of monocrotaline and of other *Senecio* alkaloids. They found that the normal value of 39·8 seconds was prolonged up to 1800 seconds in rats 24 hours after the injections of monocrotaline (120 mg./kg.).

It is of interest that in spite of the emaciation of the animals and of the haemorrhages, the values of haemoglobin (mean 92 per cent by the Sahli method) and those of total serum proteins (6·7 g./100 ml.) were rather high but still in normal limits as compared with control rats. In rats treated with isatidine various degrees of anaemia and low values of total serum proteins have been found (Schoental, 1954).

The effects of treatment of young rats with monocrotaline, when compared with those due to isatidine, and a mixture of *S. Jacobaea* alkaloids show some similarities and some marked differences. The former included the acute necrotic changes in the liver. After the treatment with monocrotaline congestion of blood vessels and the presence of thrombi in the lung and liver, and lung oedema were more prominent. On the other hand, proliferation of bile ducts, cellular infiltration round the portal systems, and endothelial proliferation of the walls of hepatic veins (Fig. 11) and of blood vessels in the lungs (Fig. 12) were a common feature in animals treated with retrorsine, isatidine, and the mixture of alkaloids from *S. Jacobaea*, Linn (Schoental, Head, and Peacock, 1954).

Till some information is available on the subject of the mechanism of action of the various *Senecio* alkaloids, attempts to explain such differences in response to them could at present be only speculative. In long term feeding experiments a prominent feature of the chronic changes was the presence of stainable iron in all the organs examined : lungs, lymph nodes, liver, spleen, kidneys. Occasionally crystals of haematoidin were present in the lungs.

In the animals in which liver biopsy was taken 6 and 13 weeks after the beginning of treatment, the livers showed *post mortem* easily recognisable sites from which the specimens were cut. The sites of liver biopsy taken at similar times from control rats were rounded and regenerated.

### SUMMARY.

Pathological changes in rats resulting from treatment with monocrotaline by skin application, by injections, and by feeding, are described, and compared with those seen in rats after treatment with other *Senecio* alkaloids.

The potential danger involved in the use, in some countries, of *Crotolaria* plants as herbal remedies, whether by ingestion, or externally by skin application, is pointed out.

### ADDENDUM

The 6 male rats in series 3C and 4 which remained healthy for over a year were then injected intraperitoneally with 12 mg. of monocrotaline. Five died and 1 was killed from 2½ to 3 months later. Two had developed liver tumours similar to those produced by the *Senecio* alkaloids described by Schoental, Head and Peacock (1954). One of these had a single tumour 5 mm. diameter of endothelial origin and the other had multiple trabecular hepatomata 2-3 mm. diameter; in this rat their was some fibrosis and much bile duct and endothelial cell proliferation. A papillary adenoma was present in the lung of a third rat.

This work has been supported by a grant from the British Empire Cancer Campaign. We are greatly indebted to Dr. Roger Adams, University of Illinois, Urbana, for a generous gift of monocrotaline, and to Dr. P. R. Peacock for his interest and criticism. Our thanks are due to Mr. S. Breslin for the photographs, to the Pathology Technical Staff for the histological sections, to Mr. C. Bern also for his part in microphotography, to Mrs. J. Jack for the protein estimations, to Mrs. J. Rae for valuable technical assistance and care of the animals, and to the Staff of the Animal House for help.

One of us (R. S.) wishes to thank the Management of the Royal Beatson Memorial Hospital and Dr. P. R. Peacock the Director of the Cancer Research Department, for kind hospitality.

### REFERENCES.

ADAMS, R., SHAFER, P. R. AND BROWN, B. H.—(1952) *J. Amer. chem. Soc.*, **74**, 5612.
CHOPRA, R. N.—(1933) ‘Indigenous Drugs of India.’ Calcutta (The Art Press).
FLYNN, F. V. AND DE MAYO, P.—(1951) *Lancet*, ii, 235.
HENRY, TH. A.—(1949) ‘The Plant Alkaloids.’ London (J. and A. Churchill Ltd.).
MARAIS, J. S. C.—(1944) *Onderstepoort J. vet. Sci.*, **20**, 61.
MONTGOMERY, G. L.—(1944) *Brit. J. Surg.*, **31**, 292.
RATNOFF, O. D. AND MIRICK, G. S.—(1949) *Johns Hopk. Hosp. Bull.*, **84**, 507.
ROSE, CH. L., FINK, R. D., HARRIS, P. N. AND CHEN, K. K.—(1945) *J. Pharmacol.*, **83**, 265.
SCHOENTAL, R.—(1954) Proc. 3rd International Congress Nutrit., Amsterdam (in press).
*Idem*, HEAD, M. A. AND PEACOCK, P. R.—(1954) *Brit. J. Cancer*, **8**, 458.
STEYN, D. G. AND DE KOCK, G.—(1932) 18*th Rep. Dir. Vet. Serv. Anim. Ind.*, p. 947.
THEILER, A.—(1920) 7*th and* 8*th Rep. Dir. Vet. Res.*, p. 56.
WATT, J. M. AND BREYER BRANDWIJK, M. G.—(1932) ‘The Medicinal and Poisonous Plants of Southern Africa.’ Edinburgh (R. and S. Livingstone).